# Extracellular Vesicles from *Candida albicans* and *Aspergillus fumigatus* Differentially Integrate PRR and cGAS-STING Signaling to Shape Macrophage Responses

**DOI:** 10.3390/jof12080575

**Published:** 2026-08-04

**Authors:** Júlia Leão Froldi, Renan E. A. Piraine, Lucas A. Tavares, Lucas Fabrício Bahia Nogueira, Patrick Santos, Fausto Almeida

**Affiliations:** 1 Departament of Biochemistry and Immunology, Ribeirão Preto Medical School, University of São Paulo, Ribeirão Preto 14049-900, São Paulo, Brazil; julialfroldi@usp.br (J.L.F.); renaneap@unifei.edu.br (R.E.A.P.); quimica.lucasbahianogueira@gmail.com (L.F.B.N.); patrickwsantos@gmail.com (P.S.); 2 Institute of Natural Resources, Federal University of Itajubá, Itajubá 37500-903, Minas Gerais, Brazil; 3 Departament of Cell and Molecular Biology, Ribeirão Preto Medical School, University of São Paulo, Ribeirão Preto 14049-900, São Paulo, Brazil

**Keywords:** fungal cells, cGAS-STING, signalling pathway, macrophages, immunomodulation

## Abstract

Fungal infections remain a major global health challenge, underscoring the need to better understand host–pathogen interactions. Extracellular vesicles (EVs) released by fungal pathogens are emerging as key modulators of immune responses. Here, we investigated how EVs from *Candida albicans* and *Aspergillus fumigatus* influence macrophage activation, focusing on the integration of pattern recognition receptors (PRRs) and cytosolic sensing pathways. EVs were characterized by nanoparticle tracking analysis, zeta potential, and cryo-electron microscopy. Human THP-1-derived macrophages were stimulated with fungal EVs, and cytokine production, gene expression, and signaling pathway activation were assessed by ELISA, RT-qPCR, and Western blotting. *C. albicans* EVs induced a strong pro-inflammatory response, with increased production of IL-1β, IL-6, TNF, IL-8, and IFN-β after 24 h. In contrast, *A. fumigatus* EVs elicited a more limited response, characterized by IL-4 production and minimal pro-inflammatory cytokine induction. Both EV populations modulated PRR expression and activated the cGAS–STING–IRF3 axis; however, *C. albicans* EVs promoted coordinated activation of TLR4, TLR9, and Dectin-1–CARD9 signaling, whereas *A. fumigatus* EVs downregulated CARD9 and dampened inflammation. These findings demonstrate that fungal EVs differentially shape macrophage responses through species-specific integration of immune sensing pathways.

## 1. Introduction

Fungal infections represent a major global public health concern [1]. Their incidence has increased over recent decades, driven by the growing number of immunocompromised individuals, widespread antimicrobial use, and the emergence of resistant fungal species [2,3]. Clinical management remains a challenge due to antifungal toxicity and the biological similarity between fungal and human cells [4].

*Candida albicans* is an opportunistic yeast that colonizes mucosal surfaces and can cause both superficial and systemic infections, particularly under conditions of immune imbalance [5,6]. In contrast, *A. fumigatus* is a filamentous environmental fungus whose airborne asexual conidia are capable of reaching the lower respiratory tract upon inhalation, leading to aspergillosis with manifestations ranging from allergic to invasive disease depending on host immune status [7,8,9]. As observed for *C. albicans*, the pathogenicity of *A. fumigatus* is strongly influenced by host immunity and its ability to modulate immune responses through the release of diverse virulence-associated factors [7,10].

In this context, intercellular communications between fungi and host cells play a critical role in determining infection outcomes. Among the mechanisms involved, extracellular vesicles (EVs) have emerged as key mediators of fungal pathogenicity and host–pathogen interactions [11]. EVs are lipid bilayer-enclosed structures released by all living cells, carrying a diverse cargo of proteins, lipids, nucleic acids, and other bioactive molecules that reflect the composition of the originating cell [12,13]. Their biogenesis involves both endosomal pathways, through multivesicular body formation, and direct budding from the plasma membrane, enabling the incorporation and transport of complex molecular cargo across the fungal cell wall into the extracellular environment [14,15]. Given these properties, fungal EVs have been increasingly investigated for their roles in cellular physiology and infection. Notably, their cargo includes virulence-associated molecules and pathogen-derived components that can be recognized by immune cells through pattern recognition receptors (PRRs), highlighting their potential to modulate innate immune responses [15,16,17,18].

Fungal EVs contain a diverse repertoire of structural and cytoplasmic components that support their capacity to engage immune cells. Increasing evidence indicates that EVs from different fungal species can actively modulate host immune responses. EVs derived from *Candida* spp. and *Cryptococcus* spp. have been shown to stimulate Toll-like receptors (TLRs), promoting the activation of intracellular signaling cascades and the production of pro-inflammatory cytokines [17,19,20]. Similarly, EVs from *A. fumigatus* interact with immune cells, including macrophages and neutrophils, inducing cytokine responses [21].

Recent studies have further expanded the role of fungal EVs in host–pathogen interactions by demonstrating their immunomodulatory activity in thermally dimorphic fungi. EVs from *Histoplasma capsulatum* modulate macrophage and dendritic cell functions, alter cytokine production, and promote fungal persistence, supporting their contribution to fungal virulence [22]. In contrast, EVs from *Talaromyces marneffei* contain immunogenic and virulence-associated molecules that promote macrophage activation, M1 polarization, increased phagocytosis, and enhanced antifungal activity [23]. Collectively, these studies demonstrate that fungal EVs are active mediators of host–pathogen interactions and that their immunological effects are species dependent, likely reflecting differences in their molecular composition.

The ability of fungal EVs to modulate innate immune responses suggests that their cargo contains pathogen-associated molecular patterns (PAMPs), including cell wall-associated glycoconjugates, proteins, lipids, and nucleic acids, capable of engaging host pattern recognition receptors (PRRs) [16,24]. Proteomic studies have further demonstrated that the molecular cargo of fungal EVs differs among species. For example, EVs from *C. albicans* are enriched in cell wall remodeling proteins and adhesins [25], whereas *A. fumigatus* EVs contain allergens, antioxidant enzymes, and other virulence-associated proteins [26]. However, the specific EV-associated molecules responsible for triggering distinct macrophage responses remain poorly defined, as do the receptors and signaling pathways that integrate these signals.

Innate immune responses to fungal pathogens are initiated by cells such as macrophages, neutrophils, and dendritic cells, which recognize PAMPs through pattern recognition receptors (PRRs), including TLRs and C-type lectin receptors (CLRs) [27,28,29]. TLR2 and TLR4 detect fungal cell wall components, whereas TLR9 senses internalized nucleic acids [30]. In parallel, CLRs such as Dectin-1 recognize carbohydrate structures, including β-glucans, and signal through the SYK-CARD9 axis, contributing to inflammatory cytokine production [30,31,32]. In addition to membrane-bound receptors, cytosolic sensors such as cGAS (cGMP-AMP synthase) detect nucleic acids and promote activation of STING (Stimulator of Interferon Genes), leading to IRF3-dependent type I interferon responses [33]. Although these pathways have been extensively characterized in response to whole fungal cells, how they are co-engaged and functionally integrated upon stimulation with fungal EVs remains unclear. Understanding this integration is essential to define how EVs shape macrophage activation and downstream immune outcomes.

EVs have emerged as key mediators of fungal communication, both between fungal cells and in interactions with host cells, underscoring their relevance in infection. Understanding how these structures are recognized by innate immune cells and how they shape downstream responses is essential for elucidating mechanisms of fungal pathogenesis, particularly in infections caused by *C. albicans* and *A. fumigatus*. Here, we investigated how EVs from *C. albicans* (Ca EVs) and *A. fumigatus* (Af EVs) modulate macrophage responses using THP-1 human macrophage-like cells. We focused on defining cytokine production profiles, modulation of PRRs, and activation of downstream signaling pathways. We hypothesized that fungal EVs act as integrative signaling platforms that differentially engage surface and cytosolic sensing pathways, resulting in species-specific macrophage activation programs. This integrative perspective provides a framework to understand how fungal EVs shape distinct immune outcomes.

## 2. Materials and Methods

### 2.1. Strains and Growth Conditions

*Candida albicans* ATCC64548 was cultured in Sabouraud dextrose broth for 24 h at 37 °C under orbital shaking. *Aspergillus fumigatus* was grown on YAG agar medium supplemented with malt extract (2% *w*/*v* glucose, 0.2% *w*/*v* yeast extract, 2% *w*/*v* malt extract, 2% *w*/*v* agar, 0.1% *v*/*v* trace elements) for 7 days at 37 °C.

### 2.2. Extracellular Vesicle (EV) Isolation

EVs were isolated as previously described by Reis et al. (2019) [34]. For *C. albicans*, 3.5 × 10^7^ cells were collected and plated onto Sabouraud agar for 24 h at 37 °C. Cells were recovered, resuspended in sterile phosphate-buffered saline (PBS), and subjected to sequential centrifugation: 5000× *g* for 15 min and 15,000× *g* for 15 min to remove cells and debris. The supernatant was filtered through 0.45 μm membranes and ultracentrifuged at 100,000× *g* for 1 h at 4 °C (Optima MAX-XP, Beckman Coulter, Brea, CA, USA). The EV pellet was resuspended in sterile PBS and stored for downstream analyses following MISEV guidelines [35]. For *A. fumigatus*, biomass was resuspended in PBS and filtered through Miracloth prior to the same centrifugation and ultracentrifugation steps. All downstream experiments using EV preparations were performed with at least three independent biological replicates derived from separate EV isolations.

### 2.3. Nanoparticle Tracking Analysis (NTA)

EV size distribution and concentration were determined using a Nanosight NS3000 system (Malvern Panalytical, Malvern, UK) equipped with NTA software v3.0. Samples were diluted 1:100 in PBS to achieve 20–100 particles per frame. Data were acquired with camera level ≥14 and analyzed using OriginPro 2024 [36].

### 2.4. Zeta Potential Measurement

Zeta potential was measured using a Zetasizer Nano ZS (Malvern Panalytical) at 25 °C. Samples were analyzed in PBS (pH 7.4) using DTS1070 cuvettes (Malvern Panalytical). Electrophoretic mobility was converted to zeta potential using the Smoluchowski model. Each sample was measured in triplicate.

### 2.5. Cryo-Electron Microscopy (Cryo-EM)

EV morphology was evaluated by Cryo-EM. Approximately 1 × 10^12^ EVs (≤4 μL), as determined by NTA, were applied to glow-discharged Quantifoil R1.2/1.3 300-mesh copper grids. Vitrification was performed using a Vitrobot Mark IV (Thermo Fisher Scientific, Waltham, MA, EUA) at 4 °C and 100% humidity. Samples were plunge-frozen in liquid ethane and imaged using a Talos Arctica microscope at 200 kV (Thermo Fisher Scientific) at the Brazilian Nanotechnology National Laboratory (LNNano/CNPEM/MCTI, Campinas, Brazil).

### 2.6. THP-1 Cell Culture and Differentiation

THP-1 monocytes (ATCC TIB-202^TM^), a widely used and reproducible model for studies of innate immune responses to microbial and fungal stimuli [37], were cultured in RPMI-1640 supplemented with 10% fetal bovine serum, 1% penicillin–streptomycin, and antimycoplasma reagent at 37 °C with 5% CO_2_. Cells with viability > 90% were seeded at 1 × 10^6^ cells/well in 6-well plates and differentiated into macrophages using 10 ng/mL PMA for 24 h.

### 2.7. Stimulation with Fungal EVs

Differentiated THP-1 macrophages were incubated under the following conditions: (i) RPMI-1640 (negative control); (ii) lipopolysaccharide (LPS, 1 ng/mL) plus Pam3Cys-Ser-(Lys)4 (PAM, 1 ng/mL), as positive control; (iii) *C. albicans* EVs; *A. fumigatus* VEs. The LPS/PAM combination was included to verify the overall inflammatory responsiveness of macrophages under the experimental conditions and was not intended to assess activation of individual PRRs. EVs were quantified by NTA and added to cells based on particle number, using a ratio of 1:1000 (cell:EVs). Cells were incubated for 6 h or 24 h at 37 °C with 5% CO_2_. Supernatants were collected, and cells were processed for RNA or protein extraction.

### 2.8. Cytokine Quantification

Cytokines (IL-4, IL-10, IL-6, IL-8, TNF, IL-1β, IL-23, and IFN- β) were quantified in supernatants by ELISA using commercial kits (BD Biosciences, San Jose, CA, USA and R&D Systems, Minneapolis, MN, USA) according to the manufacturer’s instructions. Absorbance was measured at 450 nm using a SpectraMax 190 microplate reader (Molecular Devices, San Jose, CA, USA).

### 2.9. RT-qPCR

Total RNA was extracted using the RNeasy Mini Kit (Qiagen, Hilden, Germany). RNA concentration and purity were assessed using a NanoDrop One spectrophotometer (Thermo Fisher Scientific), and only samples presenting A260/280 and A260/230 ratios between 2.0 and 2.2, with RNA concentrations ranging from 50 to 500 ng/µL, were used for subsequent analyses. cDNA was synthesized using the High-Capacity cDNA Reverse Transcription Kit (Applied Biosystems, Foster City, CA, USA). An amount of cDNA corresponding to 50 ng of input RNA was used in each qPCR reaction. Quantitative PCR was performed using SyGreen Mix (PCR Biosystems, London, UK) in a final reaction volume of 10 µL per well in a StepOnePlus Real-Time PCR System (Applied Biosystems). Amplicon specificity was assessed by post-amplification melting curve analysis using StepOne™ Software v2.3 (Applied Biosystems). Cycling conditions were 95 °C for 2 min, followed by 40 cycles of 95 °C for 5 s and 60 °C for 30 s. Gene expression of *TLR2*, *TLR4*, *TLR9*, *CLEC7A* (Dectin-1), *SYK*, *CARD9*, *MYD88*, and *NF*-*κB* was analyzed using previously validated primer pairs (Supplementary Material Table S1). Expression levels were normalized to *ACTB* (β-actin) as the endogenous reference gene, and unstimulated cells were used as the calibrator. Relative gene expression was calculated using the 2^−ΔΔCt^ method [38] and expressed as log_2_ fold change. All RT-qPCR experiments were performed using two independent biological replicates (*n* = 2). Statistical significance between unstimulated and stimulated conditions was determined by two-way ANOVA, with *p* < 0.05 considered statistically significant.

### 2.10. Western Blotting

Cells were lysed in buffer containing Tris-HCl, NaCl, glycerol, EDTA, and Triton X-100 with protease inhibitors. Protein concentration was determined by the Bradford assay, and equal amounts of protein (50 μg) were loaded per lane. Proteins were separated on SDS-PAGE gels and transferred onto nitrocellulose membranes. Equal protein loading and transfer efficiency were verified by Ponceau S staining before membrane blocking. Membranes were blocked for 1 h in 5% non-fat dry milk in TBS-T and incubated overnight at 4 °C with the respective primary antibodies (STAT1, TBK1, IRF3, IKKβ, NF-κB, cGAS, STING, p-STING, p-IRF3, p-TBK1, and GAPDH). HRP-conjugated secondary antibodies were incubated for 1 h at room temperature, and immunoreactive bands were detected using enhanced chemiluminescence in a ChemiDoc Imaging System (Bio-Rad, Hercules, CA, USA). GAPDH was used as the endogenous loading control where indicated. Western blot analyses were performed using at least three independent biological replicates, and representative blots are shown in the figures.

### 2.11. Statistical Analysis

Statistical analyses were performed using GraphPad Prism v9.0. Data are presented as mean ± SEM. Differences between groups were evaluated using one-way or two-way ANOVA followed by appropriate post hoc tests (e.g., Dunnett’s test). A *p*-value < 0.05 was considered statistically significant.

## 3. Results

### 3.1. Characterization of Fungal EVs

**Figure 1:**
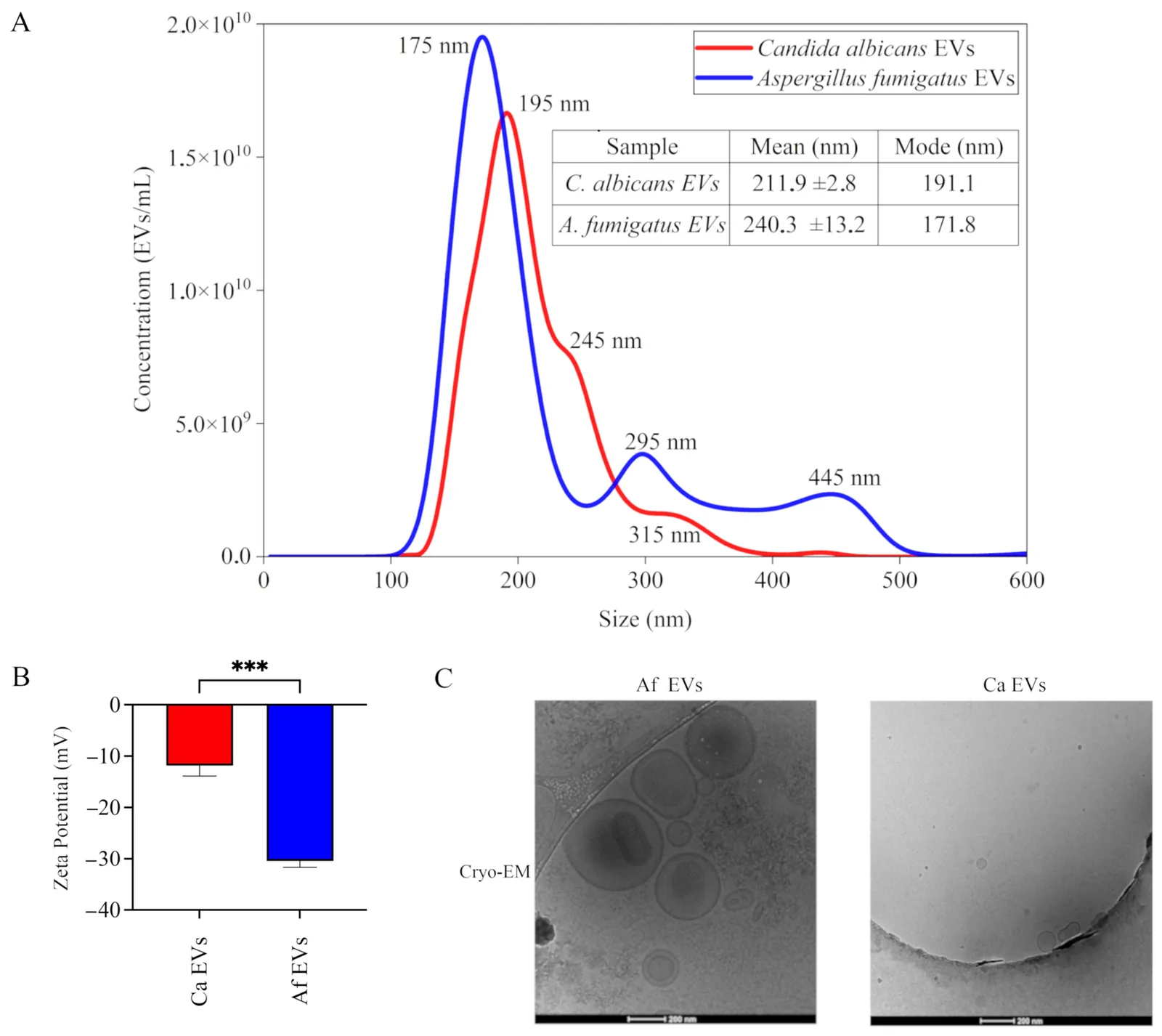
Characterization of extracellular vesicles (EVs) isolated from *A. fumigatus* (Af) and *C. albicans* (Ca). (**A**) Size distribution and particle concentration of EVs determined by nanoparticle tracking analysis (NTA). Curves represent the particle profile as a function of hydrodynamic diameter (nm); graphs were generated using OriginLab^®^ (Version 2026b). The inset highlights differences in mean and mode values between species. (**B**) Zeta potential (mV) of Ca EVs and Af EVs, demonstrating a significant difference in surface charge between groups (*** *p* < 0.001). (**C**) Morphological characterization of EVs by cryo-electron microscopy (cryo-EM). Representative images showing predominantly spherical vesicles. Cryo-EM was used to confirm EV morphology and structural integrity, whereas the overall size distribution of the EV population was determined by NTA.

EVs isolated from *A. fumigatus* and *C. albicans* were characterized to confirm sample quality and consistency prior to downstream analyses. Nanoparticle tracking analysis (NTA) revealed heterogeneous size distributions for both EV populations, with a mean diameter of 240.3 ± 13.2 nm for Af EVs and 211.9 ± 2.8 nm for Ca EVs (Figure 1A). Modal size analysis indicated peak populations at 171.8 nm for Af EVs and 191.1 nm for Ca EVs. Cryo-EM confirmed the presence of predominantly spherical EVs with preserved morphology (Figure 1C). The overall EV size distribution was determined exclusively by NTA, whereas the cryo-EM images represent only a small subset of vesicles and were included for morphological characterization.

Zeta potential measurements demonstrated distinct surface charge properties between the EV populations. Ca EVs exhibited an average zeta potential of −11 mV, whereas Af EVs exhibited a more negative value of −30 mV (Figure 1B), indicating differences in surface properties that may influence vesicle stability and interactions with host cells. Together, these data confirm that both EV preparations are structurally intact and suitable for downstream functional analyses.

### 3.2. EVs Derived from C. albicans and A. fumigatus Differentially Modulate Early Macrophage Cytokine Production

**Figure 2:**
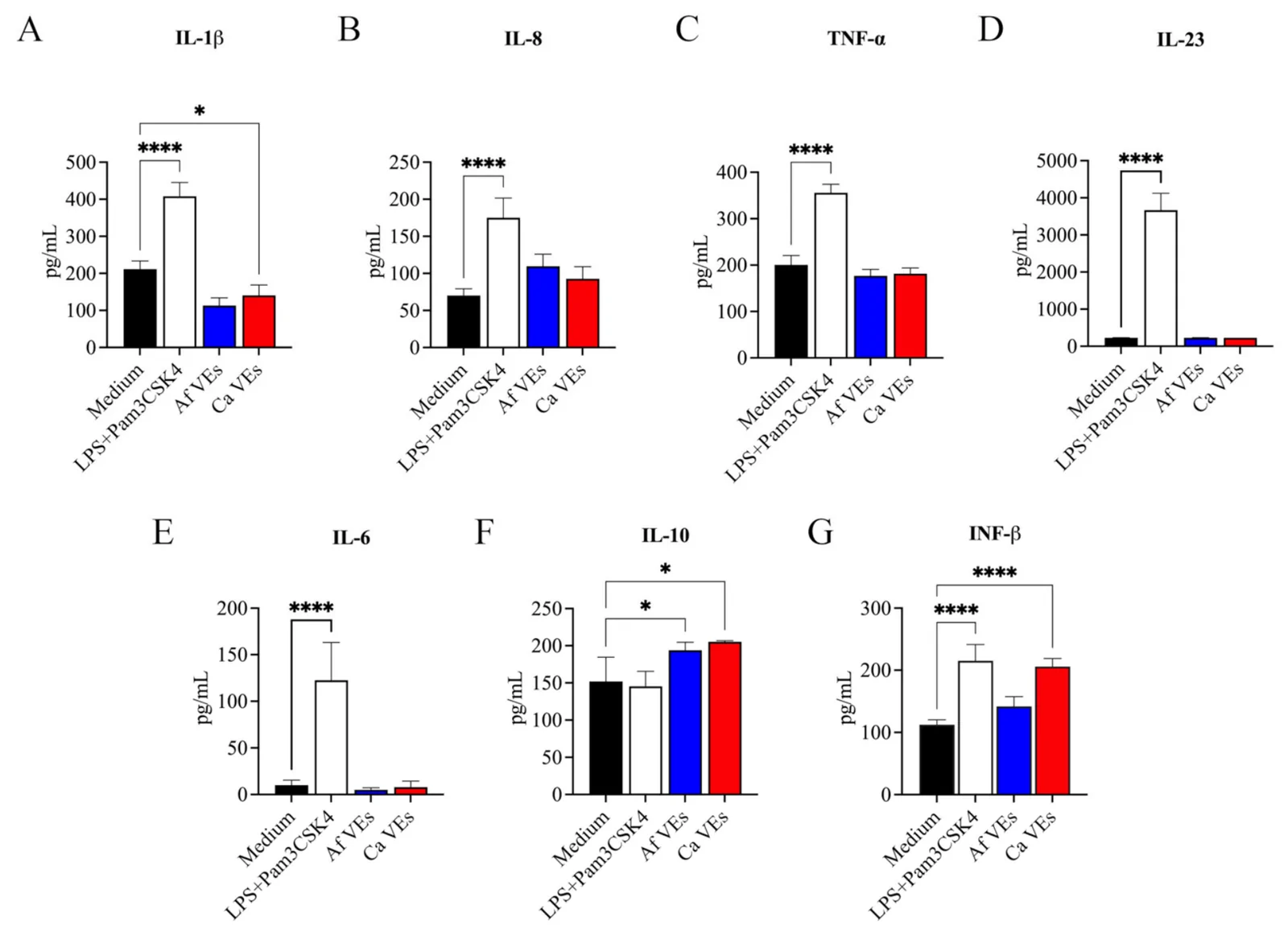
Cytokine production by THP-1-derived macrophages stimulated with extracellular vesicles (EVs) from *C. albicans* (Ca) and *A. fumigatus* (Af). THP-1 macrophages were stimulated for 6 h with EVs derived from Ca or Af. Cytokine levels were quantified in culture supernatants and are represented as pg/mL: IL-1β (**A**), IL-8 (**B**), TNF-α (**C**), IL-23 (**D**), IL-6 (**E**), IL-10 (**F**), and IFN-β (**G**). Data are expressed as mean ± SEM and were analyzed by one-way analysis of variance (ANOVA) followed by Dunnett’s multiple comparisons test against the control group. Statistical significance is indicated as follows: **** *p* < 0.0001 and * *p* < 0.05.

Cytokine production by THP-1 macrophages was evaluated after 6 h and 24 h of stimulation with fungal EVs. At 6 h, EVs from both *C. albicans* and *A. fumigatus* induced minimal changes in cytokine production, with no significant increase in pro-inflammatory cytokines detected (Figure 2A–G). Notably, a reduction in IL-1β levels was observed in response to EV stimulation (Figure 2A). At this early time point, EVs primarily elicited subtle immunomodulatory effects, characterized by modest changes in IL-10 and IFN-β levels, particularly in cells stimulated with Ca EVs (Figure 2F,G).

**Figure 3:**
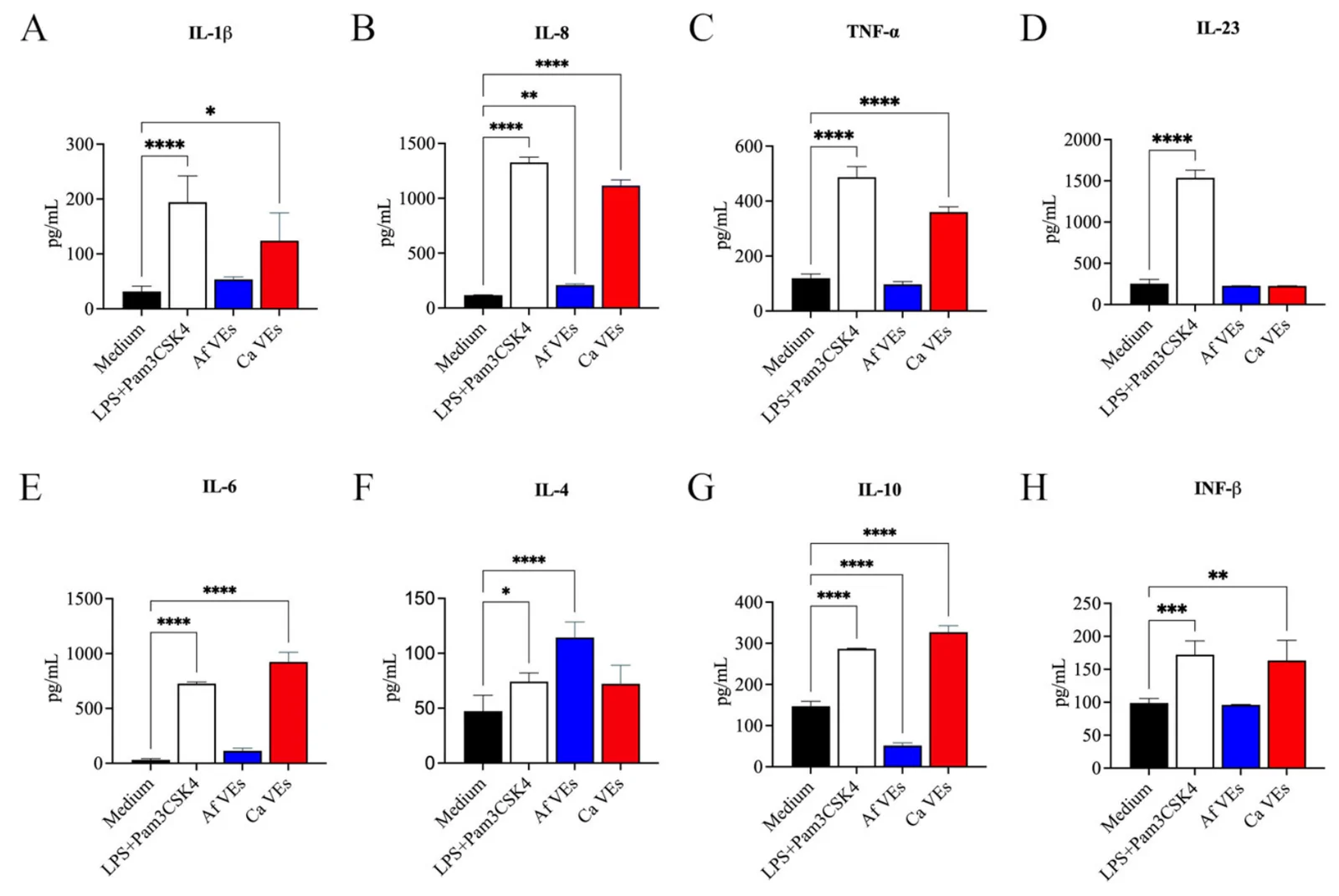
Cytokine production by THP-1-derived macrophages stimulated with extracellular vesicles (EVs) from *C. albicans* (Ca) and *A. fumigatus* (Af) for 24 h. THP-1 macrophages were stimulated for 24 h with EVs derived from Ca or Af. Cytokine concentrations in culture supernatants were measured and are presented as pg/mL: IL-1β (**A**), IL-8 (**B**), TNF-α (**C**), IL-23 (**D**), IL-6 (**E**), IL-4 (**F**), IL-10 (**G**), and IFN-β (**H**). Data are shown as mean ± SEM and were analyzed using one-way analysis of variance (ANOVA) followed by Dunnett’s multiple comparisons test against the control group. Statistical significance is indicated as follows: **** *p* < 0.0001, *** *p* < 0.001, ** *p* < 0.01, and * *p* < 0.05.

In contrast to the limited early response, stimulation for 24 h revealed a marked divergence between EVs from *C. albicans* and *A. fumigatus*. Macrophages exposed to Ca EVs exhibited a robust pro-inflammatory profile, characterized by significantly increased production of IL-6, IL-8, IL-1β, and TNF-α (Figure 3A–C,E). In contrast, Af EVs failed to induce a comparable pro-inflammatory response, with no significant increase observed for these cytokines. Regarding regulatory cytokines, IL-10 levels were significantly elevated following stimulation with Ca EVs (Figure 3G), whereas IL-4 was selectively increased in macrophages treated with Af EVs (Figure 3F), indicating a distinct immunomodulatory profile. Consistent with these differences, IFN-β levels were sustained and significantly upregulated exclusively in response to Ca EVs at 24 h, while a slight reduction was observed in cells stimulated with Af EVs (Figure 3H). Together, these findings indicate that EVs from *C. albicans* and *A. fumigatus* drive fundamentally distinct macrophage activation programs.

### 3.3. EVs from C. albicans and A. fumigatus Differentially Modulate PRR and Cytosolic Signaling Pathways in THP-1 Macrophages

**Figure 4:**
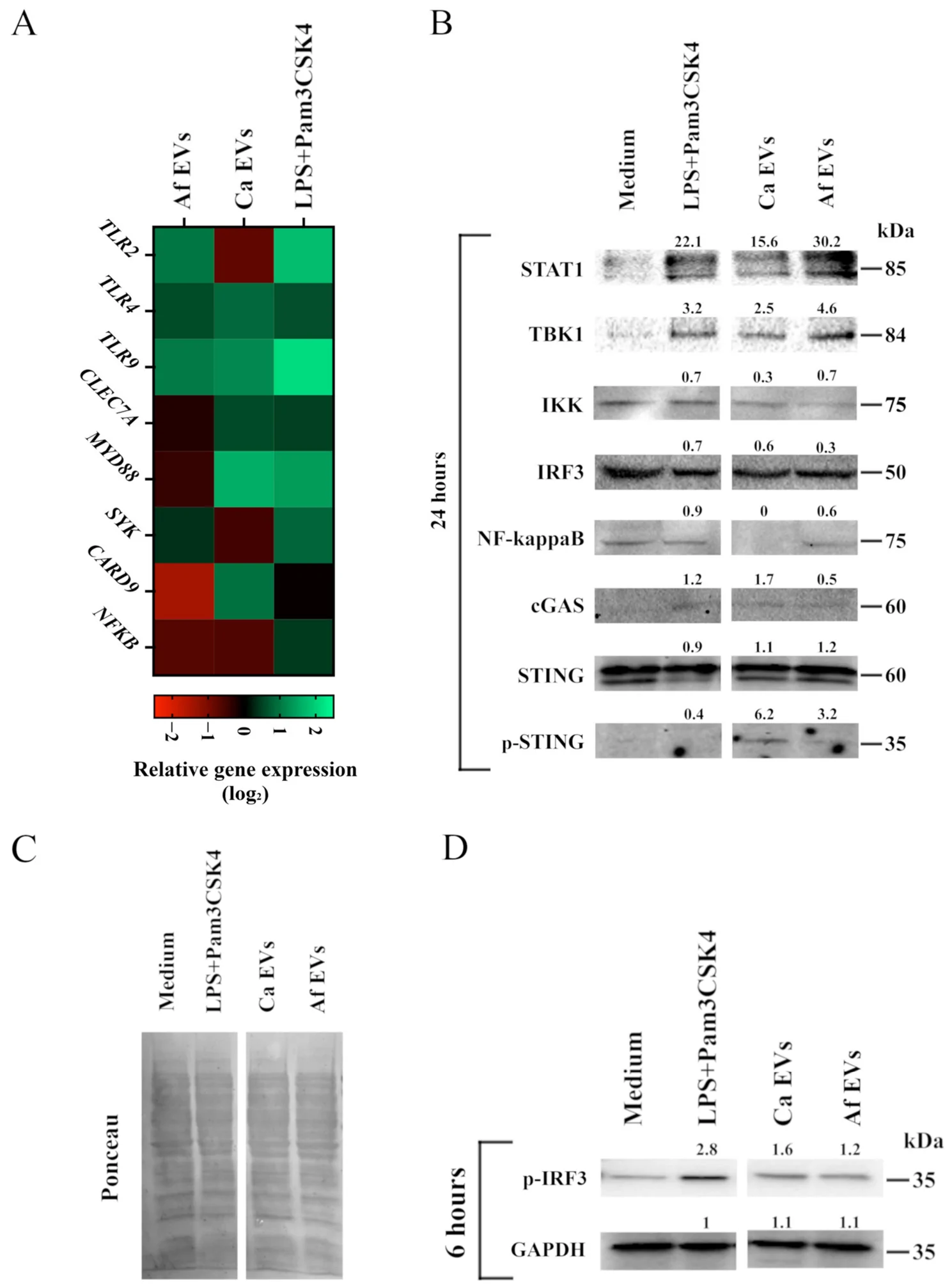
Modulation of pattern recognition receptors (PRRs) and intracellular signaling pathways in THP-1-derived macrophages stimulated with extracellular vesicles (EVs). (**A**) Heatmap showing relative gene expression (log_2_) encoding PRRs and signaling-associated proteins (*TLR2*, *TLR4*, *TLR9*, *CLEC7A*, *MYD88*, *SYK*, *CARD9*, and *NF-κB*) following stimulation with EVs. Green indicates up-regulation and red indicates down-regulation relative to basal expression, normalized to the housekeeping gene *ACT1*. (**B**) Western blot analysis of STAT1, TBK1, IKK, IRF3, NF-κB, cGAS, STING, and phosphorylated STING (p-STING) after 24 h of stimulation with EVs from *C. albicans* (Ca) and *A. fumigatus* (Af). (**C**) Ponceau S staining used as a loading control for protein normalization in the 24 h experiments. (**D**) Representative Western blot showing IRF3 phosphorylation (p-IRF3) after 6 h of stimulation with fungal EVs. LPS + Pam3CSK4 was used as a positive control. GAPDH served as an endogenous control for normalization. Relative protein quantification is shown in the figure, normalized to the control group (medium), which was set to 1.

To investigate the mechanisms underlying the cytokine responses induced by fungal EVs, we evaluated the modulation of key receptors and signaling pathways. Gene expression analysis revealed distinct patterns between EVs from *A. fumigatus* and *C. albicans* (Figure 4A and Supplementary Material Figure S1). Af EVs induced increased expression of *TLR2*, *TLR4*, and *TLR9*, while *CLEC7A* (Dectin-1) remained unchanged. In contrast, Ca EVs promoted upregulation of *TLR4*, *TLR9*, and *CLEC7A*, accompanied by a reduction in *TLR2* expression. Despite modulation of TLR expression, *MYD88* levels remained unchanged, while *NF-κB* expression was reduced in response to EVs from both species. Within the CLR pathway, SYK expression was not significantly altered; however, *CARD9* exhibited species-dependent regulation, with increased expression in response to Ca EVs and downregulation following stimulation with Af EVs.

We next assessed key signaling proteins by Western blot (Figure 4B and Supplementary Material Figure S2). Consistent with the transcriptional data, components of the classical NF-κB pathway showed limited activation. IKK levels were slightly reduced following stimulation with Ca EVs and remained near basal levels in response to Af EVs. Accordingly, NF-κB levels were decreased in cells treated with Af EVs and were undetectable after stimulation with Ca EVs.

Analysis of cytosolic sensing pathways revealed the cGAS–STING axis. Both EV populations were associated with increased levels of total STING and phosphorylated STING (p-STING). In contrast, cGAS expression displayed species-specific regulation, with increased levels in response to Ca EVs and reduced levels following stimulation with Af EVs. TBK1, a kinase involved in IRF3 activation, was elevated in response to EVs from both species. Although total IRF3 levels were reduced, early time-point analysis (Figure 4D) indicated increased phosphorylation of IRF3 (p-IRF3), supporting pathway engagement. In line with these findings, Western blot analysis consistently showed higher STAT1 immunoreactivity following stimulation with EVs from both fungal species (Figure 4B), consistent with activation of IFN-associated signaling pathways. Together, these data indicated that fungal EVs differentially coordinated PRR and cytosolic sensing pathways, contributing to the distinct macrophage responses observed.

## 4. Discussion

Fungal EVs have emerged as important mediators of host–pathogen communication [11]; however, how they integrate multiple immune sensing pathways remains incompletely understood. Here, we demonstrated that EVs from *A. fumigatus* and *C. albicans* differentially coordinate PRR and cytosolic signaling pathways, resulting in distinct macrophage activation programs.

EVs from both species displayed size distribution and morphology consistent with previous reports [17,18,39]. Both populations exhibited heterogeneous size profiles centered around ~200 nm and typical spherical morphology, as confirmed by NTA and cryo-EM analyses. Differences in surface charge between EVs may influence vesicle stability and interactions with host cells, potentially contributing to the species-specific immune response observed [39].

In addition to size and morphology, EVs exhibited distinct surface charge properties, as indicated by differences in zeta potential. Variations in vesicle charge may influence colloidal stability and interactions with host cells, potentially affecting uptake and persistence under physiological conditions [20,40,41]. Following structural characterization, we investigated the immunomodulatory potential of fungal EVs. Macrophages are key effector cells in antifungal immunity, recognizing pathogen-associated molecular patterns (PAMPs) through PRRs and initiating signaling pathways that drive inflammatory responses [42]. In this context, fungal EVs may contribute to host–pathogen interactions by delivering bioactive cargo that engages innate immune sensing mechanisms.

Our findings demonstrated that EVs from *A. fumigatus* and *C. albicans* elicit distinct response profiles in THP-1 macrophages, indicating that these vesicles act as active modulators of inflammation rather than passive carriers of fungal components. THP-1-derived macrophages were used as the experimental model because they are widely employed to investigate innate immune responses to microbial and fungal stimuli [17,18,20,23,37]. This model provides a homogeneous genetic background and high experimental reproducibility while minimizing the donor-to-donor variability inherent to primary human monocyte-derived macrophages [37], hereby providing a robust and reproducible platform for comparing the immunomodulatory effects induced by Ca and Af EVs.

In the present study, fungal EVs were characterized in terms of size distribution (NTA) and morphology (cryo-EM), but their molecular cargo (e.g., proteins, lipids, and nucleic acids) was not directly analyzed. Consequently, the specific EV-associated molecules responsible for the distinct macrophage responses observed remain to be identified, and future proteomic and molecular characterization of Ca EVs and Af EVs will be required to establish direct mechanistic links between EV cargo and the immune signaling pathways described here.

Ca EVs induced a robust inflammatory response, characterized by increased production of pro-inflammatory cytokines alongside IL-10, whereas Af EVs promoted a more limited and distinct profile, with increases primarily restricted to IL-4 and IL-8 after 24 h of stimulation.

At early time points (6 h), both EV populations elicited modest effects, suggesting that EV-mediated immune modulation is time-dependent. Differences in EV uptake kinetics between species may contribute to this pattern, as previous studies have shown higher internalization rates for Ca EVs compared to Af EVs [19,26], potentially explaining the stronger inflammatory response observed at later stages. Consistent with these observations, previous studies have reported IFN-β induction following stimulation with Ca EVs, whereas responses to Af EVs appear more variable across experimental systems [18,19,26].

A more pronounced inflammatory profile was observed at later time points following stimulation with Ca EVs. After 24 h, this response was characterized by a sustained increase in IL-10, accompanied by significant upregulation of pro-inflammatory cytokines, including IL-1β, IL-8, TNF-α, and IL-6, consistent with a coordinated inflammatory response [43]. In contrast, Af EVs induced a distinct profile, marked by increased IL-4 production and limited induction of pro-inflammatory cytokines, suggesting a more immunomodulatory phenotype [43]. These findings are in line with previous reports describing robust inflammatory and IFN-associated responses to Ca EVs [17], whereas responses to Af EVs appear more variable and less characterized, particularly at later time points [44]. Notably, the selective induction of IL-4 observed in our study points to a distinct immune polarization pattern that has not been extensively explored in the context of Af EVs.

The reduced pro-inflammatory response induced by Af EVs at 24 h may be related to differences in their molecular composition. Previous studies have shown that fungal EV cargo can include molecules with immunomodulatory properties, which may influence downstream signaling and cytokine production. Together, these observations suggest that while Af EVs may initiate early immune recognition, prolonged exposure favors a regulatory or alternative activation profile [43,44].

The detection of IFN-β as early as 6 h following stimulation with Ca EVs suggests the involvement of signaling pathways beyond canonical surface receptor-mediated recognition. Given that type I IFN production is closely associated with IRF3 activation downstream of cytosolic sensors or endosomal TLRs [30,31], these findings suggest that EVs may access intracellular compartments and engage nucleic acid-sensing mechanisms. This supports a role for fungal EVs as complex immunomodulatory structures capable of integrating multiple signaling pathways.

Consistent with this interpretation, we observed increased phosphorylation of IRF3 and modulation of upstream signaling components, including TBK1 and elements of the cGAS–STING pathway. Although total levels of cGAS and STING remained relatively stable, their phosphorylated forms were increased, supporting engagement of this signaling axis. These observations are in agreement with previous studies implicating cGAS–STING in the recognition of Ca EVs [18], providing additional support for cytosolic sensing of fungal EVs.

Importantly, our data further suggest that this cytosolic pathway may not be restricted to Ca EVs. Despite earlier reports not associating Af EVs with cGAS–STING activation [19], we detected phosphorylation of STING and IRF3 following stimulation with Af EVs. While these findings do not establish direct activation, they are consistent with the possibility that Af EVs also engage cytosolic sensing mechanisms, expanding the current understanding of fungal EV-host interactions.

Temporal analysis of signaling pathways revealed distinct patterns between early and late events following EV stimulation. Phosphorylation of IRF3 (p-IRF3) was detected within the first 6 h, consistent with established activation kinetics [18,44]. In contrast, phosphorylated STING (p-STING) was observed at 24 h, suggesting sustained or delayed engagement of this pathway. Although STING phosphorylation is typically reported at earlier timepoints [19,45,46], the lack of intermediate measurements in this study precludes distinction between transient and sustained activation. Nevertheless, the combined detection of early p-IRF3 and later p-STING is consistent with engagement of the cGAS-STING signaling axis.

In addition to cytosolic sensing, IRF3 activation may also be influenced by TLR signaling, particularly through the TLR4–TRIF pathway [47]. TLRs are well-established receptors for fungal cell wall PAMPs, including mannans, β-glucans, and various glycoconjugates [28], components previously identified in the proteomic and biochemical characterization of Ca and Af EVs (EVs) [26,48]. Our findings support this recognition pathway, as we observed a modulation of *TLR4* expression without a concomitant increase in *MYD88* or *NF-κB*. This divergence from the classical inflammatory cascade [47,49] suggests a signaling redirection toward MyD88-independent pathways, favoring Type I IFN induction. Although the TRIF-dependent pathway is under-explored in fungal immunology compared to other pathological contexts [50,51,52], its potential role here aligns with the established importance of TLR4 in the immune recognition of Ca EVs [53].

Notably, the downstream effects of these pathways differed between EVs from *A. fumigatus* and *C. albicans.* Ca EVs induced a molecular profile characterized by upregulation of *TLR4* and *TLR9*, together with the downregulation of *TLR2* and *MYD88*, consistent with the observed IFN-β production and pro-inflammatory cytokines [31]. In contrast, Af EVs elicited a distinct profile, with upregulation of *TLR2*, *TLR4*, and *TLR9* but no significant changes in *MYD88*, accompanied by limited induction of pro-inflammatory cytokines and an IL-4-enriched response. These findings suggest that, although both EV populations engage TLRs, the resulting signaling output is species-specific and leads to divergent macrophage activation states.

In parallel with TLR modulation, CLR signaling also exhibited species-specific patterns. In macrophages stimulated with Ca EVs, increased expression of *CLEC7A* and *CARD9* supports involvement of the Dectin-1-CARD9 axis, consistent with previous findings in fungal models [54,55]. Although *SYK* expression was not altered, this kinase is primarily regulated by phosphorylation [56], an event not assessed in this study. Nevertheless, the observed transcriptional profile, together with cytokine production, is consistent with engagement of this pathway [31,55]. In contrast, Af EVs were associated with a reduced CLR signaling profile, characterized by unchanged *CLEC7A* expression and downregulation of *CARD9* and *SYK* transcripts. Given the central role of CARD9 in Dectin-1-mediated signaling, its downregulation may contribute to the attenuated inflammatory response observed. This interpretation is supported by studies demonstrating that CARD9 deficiency impairs the production of pro-inflammatory cytokines such as TNF and IL-1β [57]. Nevertheless, further studies evaluating SYK phosphorylation will be important to confirm activation of this pathway.

In addition, stimulation with EVs from both species resulted in increased STAT1 levels, a key regulator of interferon-stimulated genes and antifungal immunity [58]. Although STAT1 phosphorylation was not directly evaluated, the increase in total protein levels suggests modulation of interferon-associated pathways. Given the established relationship between STAT1 abundance and its active phosphorylated form [58], these findings are consistent with engagement of the JAK–STAT signaling axis, although confirmation of STAT1 activation will require evaluation of its phosphorylated form.

**Figure 5:**
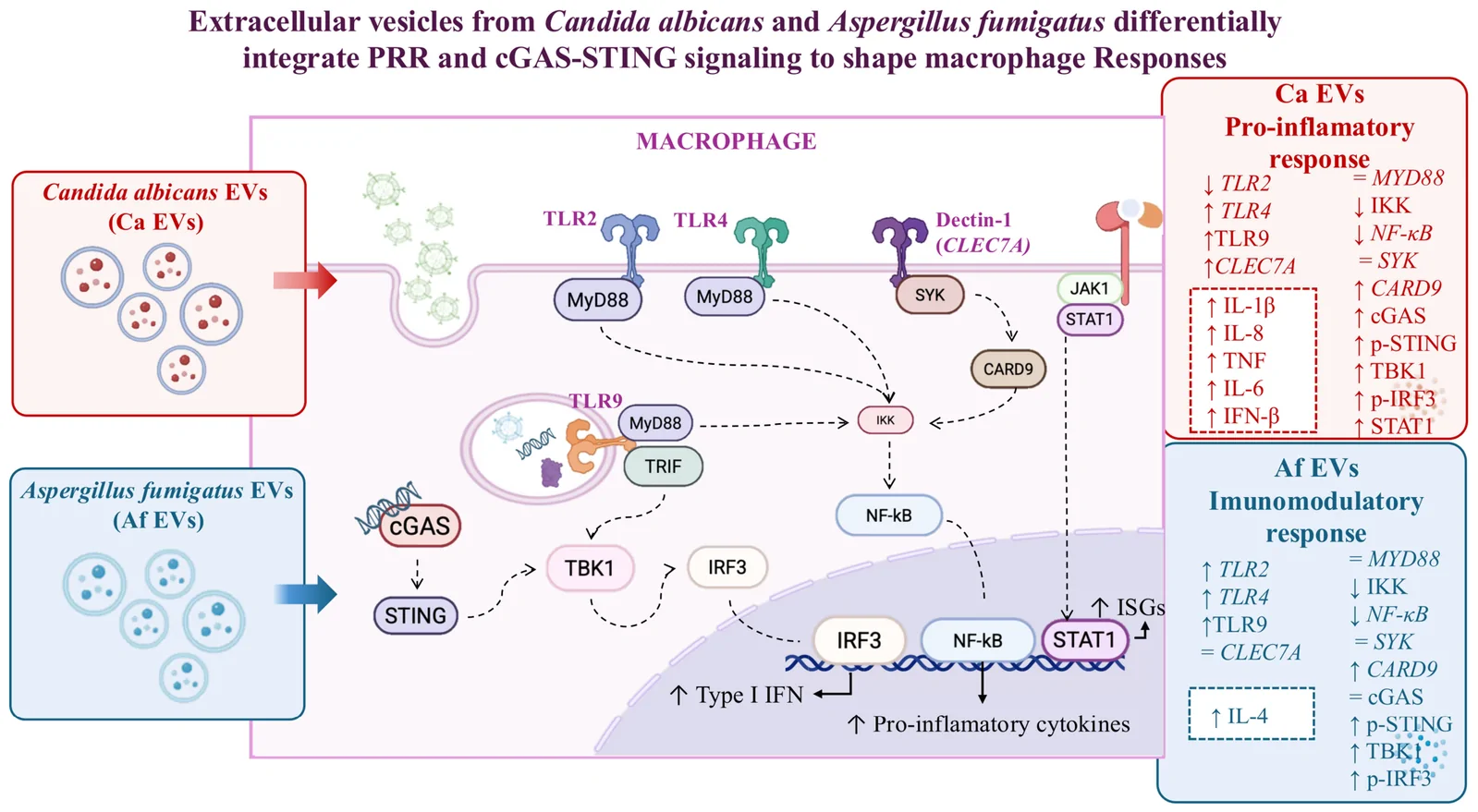
Proposed model summarizing the immunomodulatory responses induced by extracellular vesicles (EVs) from *Candida albicans* (Ca EVs) and *Aspergillus fumigatus* (Af EVs) in macrophages. The scheme summarizes the cytokine, gene expression, and signaling data obtained in this study as a proposed model of EV-mediated immune modulation. Arrows and dashed lines are used only for graphical layout and do not indicate different types of interactions or levels of evidence. Created with BioRender.com.

Together, these results indicate that fungal EV recognition involves the integration of multiple signaling pathways rather than isolated receptor activation. In Ca EVs, coordinated modulation of TLR4, TLR9, and the Dectin-1-CARD9 axis, together with engagement of STING, IRF3 and STAT1, is associated with a robust pro-inflammatory and Type I IFN response. In contrast, Af EVs, despite engaging TLRs and components of cytosolic sensing pathways, promote a distinct immunomodulatory profile, likely influenced by reduced activation of the Dectin-1-CARD9 axis. These findings highlight that the integration of surface, endosomal, and cytosolic sensing pathways determines the functional outcome of macrophage response to fungal EVs, although further studies will be important to confirm the specific contribution of individual pathways. Understanding these species-specific signaling networks provides new insight into fungal pathogenesis and potential targets for immunomodulatory strategies (Figure 5).

## Data Availability

The data presented in this study are available within the article and its Supplementary Materials.

## Supplementary Materials

The following supporting information can be downloaded at: https://www.mdpi.com/article/10.3390/jof12080575/s1, Table S1: Primer sequences used in RT-qPCR; Figure S1: Relative mRNA expression of genes associated with PRR signaling in THP-1-derived macrophages stimulated with extracellular vesicles (EVs) from *C. albicans* (Ca EVs) or *A. fumigatus* (Af EVs). Panels show the statistical analyses corresponding to the heatmap presented in Figure 4 of the main manuscript. Gene expression was determined by RT-qPCR for *TLR2* (A), *TLR4* (B), *TLR9* (C), *CLEC7A* (D), *MYD88* (E), *SYK* (F), *CARD9* (G), and *NFKB* (H). Data are presented as relative mRNA expression (log_2_ fold change) compared with unstimulated control cells. LPS + Pam3CSK4 was used as the positive control. Bars represent the mean ± SEM of two independent experiments. Statistical significance was determined by one-way ANOVA followed by Dunnett’s multiple comparisons test. *P* < 0.05 versus the unstimulated control; Figure S2: Quantitative analysis of the Western blot experiments presented in Figure 4B. Bar graphs represent the relative protein expression obtained by densitometric analysis of independent Western blot experiments; Figure S3: Full, uncropped Western blot membranes corresponding to the immunoblots presented in Figure 4B,D. Representative membranes are shown for (A) STAT1, (B) TBK1, (C) IKK, (D) IRF3, (E) NF-kB, (F) cGAS, (G) STING, (H) p-STING, (I) GAPDH, and (J) p-IRF3. References [37,59,60,61,62,63,64,65,66] are cited in the Supplementary Materials.

## Abbreviations

The following abbreviations are used in this manuscript:

EVs	Extracellular Vesicles
Af EVs	*A. Fumigatus* EVs
Ca EVs	*C. albicans* EVs
CARD9	Caspase recruitment domain-containing protein 9
cGAS	Cyclic GMP-AMP synthase
CLRs	C-type lectin
Cryo-EM	Cryo-electron microscopy
IFN	Interferon
IL	Interleukin
IRF3	Interferon regulatory factor 3
JAK	Janus Kinase
LPS	Lipopolysaccharide
Myd88	Myeloid differentiation primary response protein MyD88
NK-κB	Nuclear Factor kappa-light-chain-enhancer of activated B cells
PAMPs	Pathogen-associated molecular patterns
PRR	Pattern recognition receptors
STAT1	Signal Transducer and Activator of Transcription 1
STING	Stimulator of Interferon Genes
SYK	Spleen Tyrosine Kinase
TBK1	TANK-binding kinase 1
TLR	Toll-like receptor
TNF	Tumor necrosis factor
