# Supplementary material for: Extracellular Vesicles from Candida albicans and Aspergillus fumigatus Differentially Integrate PRR and cGAS-STING Signaling to Shape Macrophage Responses
Source: J Fungi (Basel). 2026 Aug 4;12(8):575. doi: 10.3390/jof12080575 (PMC13514852; doi:10.3390/jof12080575)
Supplement: Supplementary file 1 [file jof-12-00575-s001.zip › Supplementary Material - Review_Round2.pdf]

Supplementary Material

Fungal EVs Orchestrate Distinct Macrophage Responses Through Integrated Pattern Recognition Signaling

Júlia L. Froldi[1], Renan E. A. Piraine[1*], Lucas A. Tavares[3], Lucas P. N. Bahia[1], Patrick W. Santos[1], Fausto Almeida[1].

Table S1. Primer sequences used in RT-qPCR.

Gene	Sense sequence	Antisense sequence	Source
ACTB (β-Actin)	ATTGCCGACAGGATGCAGAA	GCTGATCCACATCTGCTGGAA	[37]
TLR2	CAGGAGCTCTTAGTGACCAAGTGA A	CACAAAGTATGTGGCATTGTCCAG	[59]
TLR4	GGTCAGACGGTGATAGCGAG	TTTAGGGCCAAGTCTCCACG	[60]
TLR9	TCTTGAAGGCCTGGTGTTGA	AAGGACAGGTTAAGCTTGCG	[61]
CLEC7A	AGCCTACCTGTAGGTCGACAA	CTGAGGTCAAGATAAATGCAGAAA	[62]
SYK	CATGGAAAAATCTCTCGGGAAGA	GTCGATGCGATAGTGCAGCA	[63]
CARD9	TCCGACCTGGAAGATGGCTCAC	CAGAGCTGCAAAGGGCTGTTT C	[64]
MYD88	GCATATGCCTGAGCGTTTCG	TTCTGATGGGCACCTGGAGA	[65]
NF-κB	TGAGTCCTGCTCCTTCCA	GCTTCGGTGTAGCCCATT	[66]

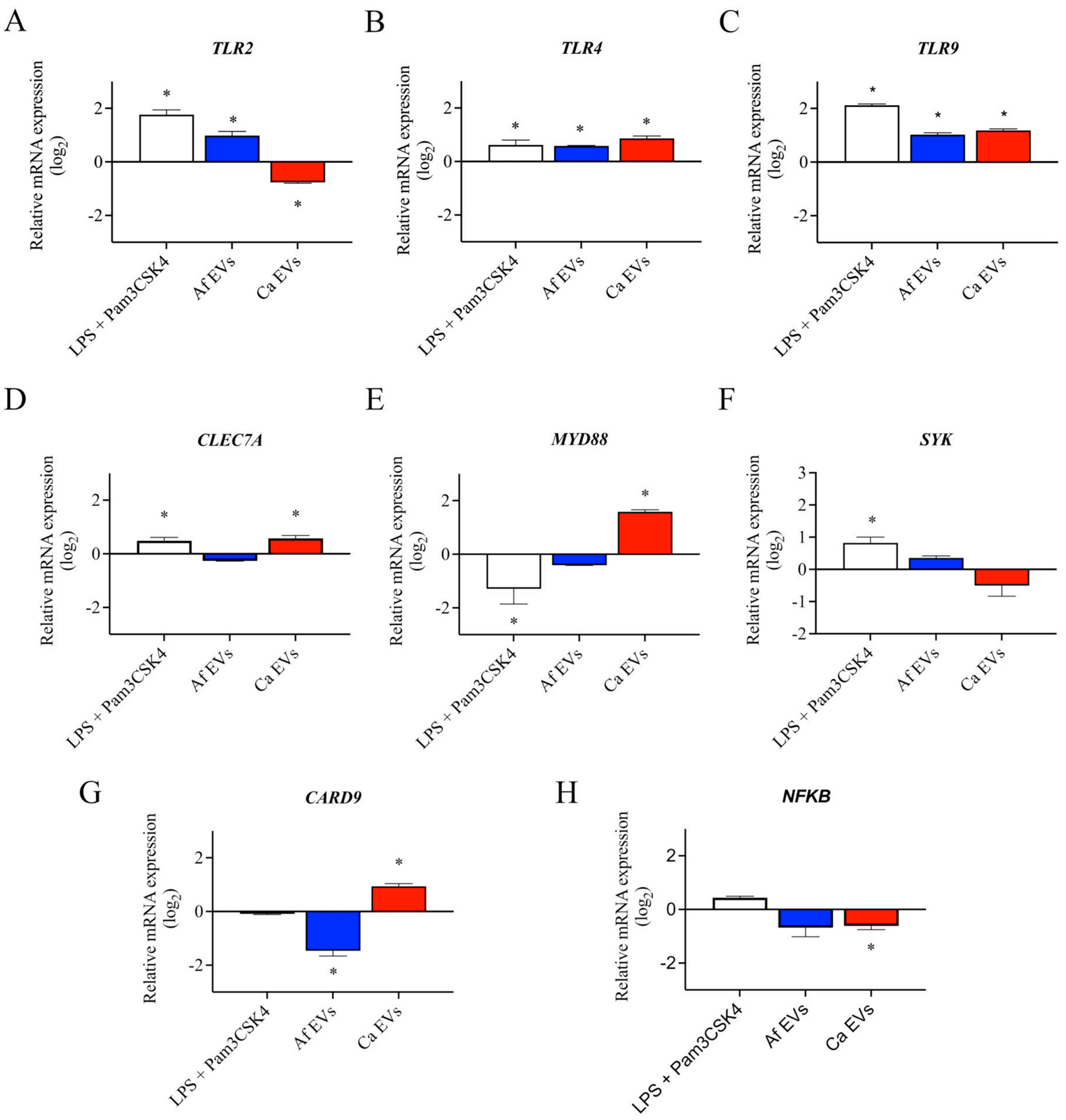

Figure S1: Relative mRNA expression of genes associated with PRR signaling in THP-1-derived macrophages stimulated with extracellular vesicles (EVs) from *C. albicans* (Ca EVs) or *A. fumigatus* (Af EVs). Panels show the statistical analyses corresponding to the heatmap presented in Figure 4 of the main manuscript. Gene expression was determined by RT-qPCR for *TLR2* (A), *TLR4* (B), TLR9 (C), CLEC7A (D), MYD88 (E), SYK (F), CARD9 (G), and NFKB (H). Data are presented as relative mRNA expression (log_2 fold change) compared with unstimulated control cells. LPS + Pam3CSK4 was used as the positive control. Bars represent the mean ± SEM of two independent experiments. Statistical significance was determined by one-way ANOVA followed by Dunnett's multiple comparisons test. $P < 0.05$ versus the unstimulated control.

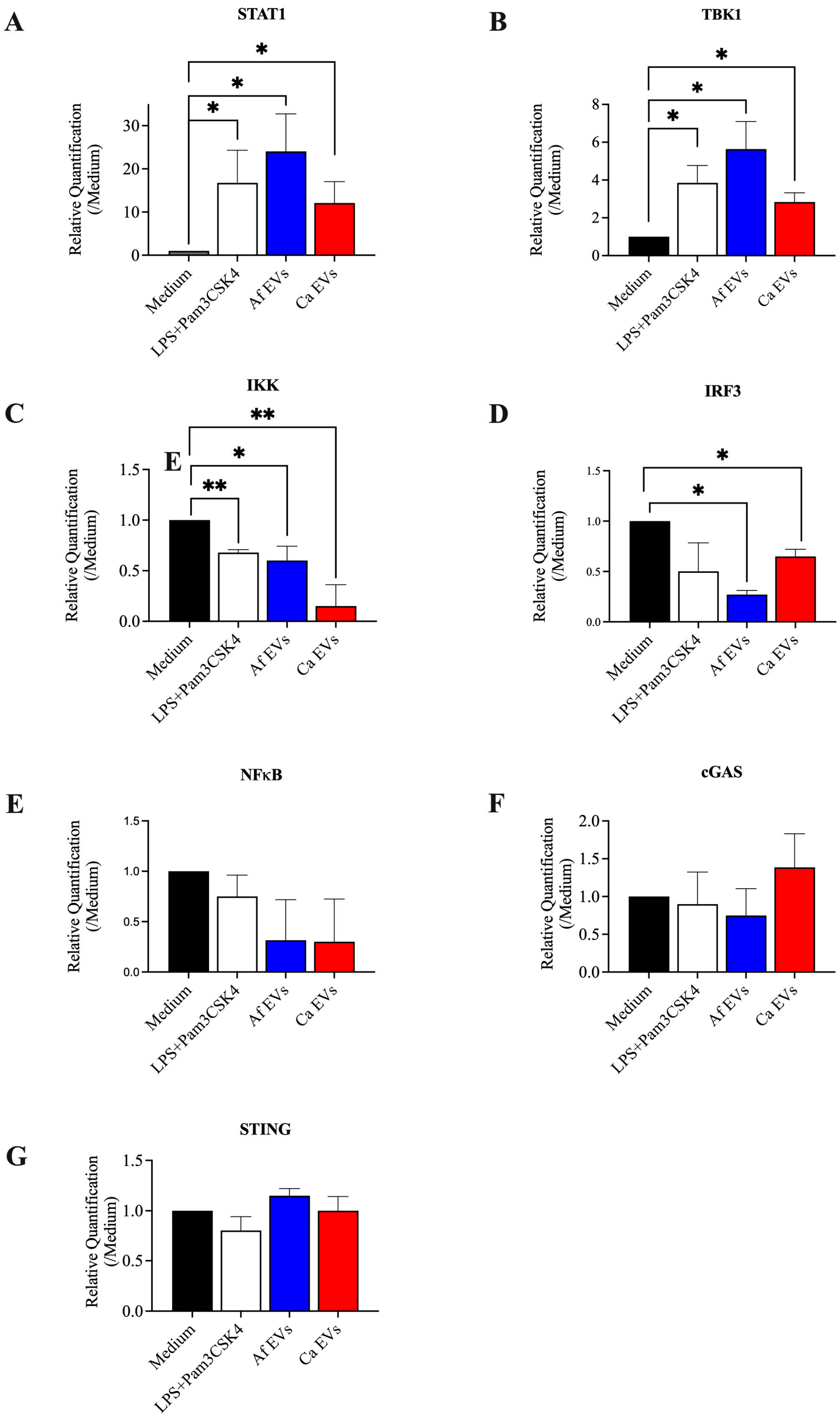

Figure S2: Quantitative analysis of the Western blot experiments presented in Figure 4B. Bar graphs represent the relative protein expression obtained by densitometric analysis of independent Western blot experiments.

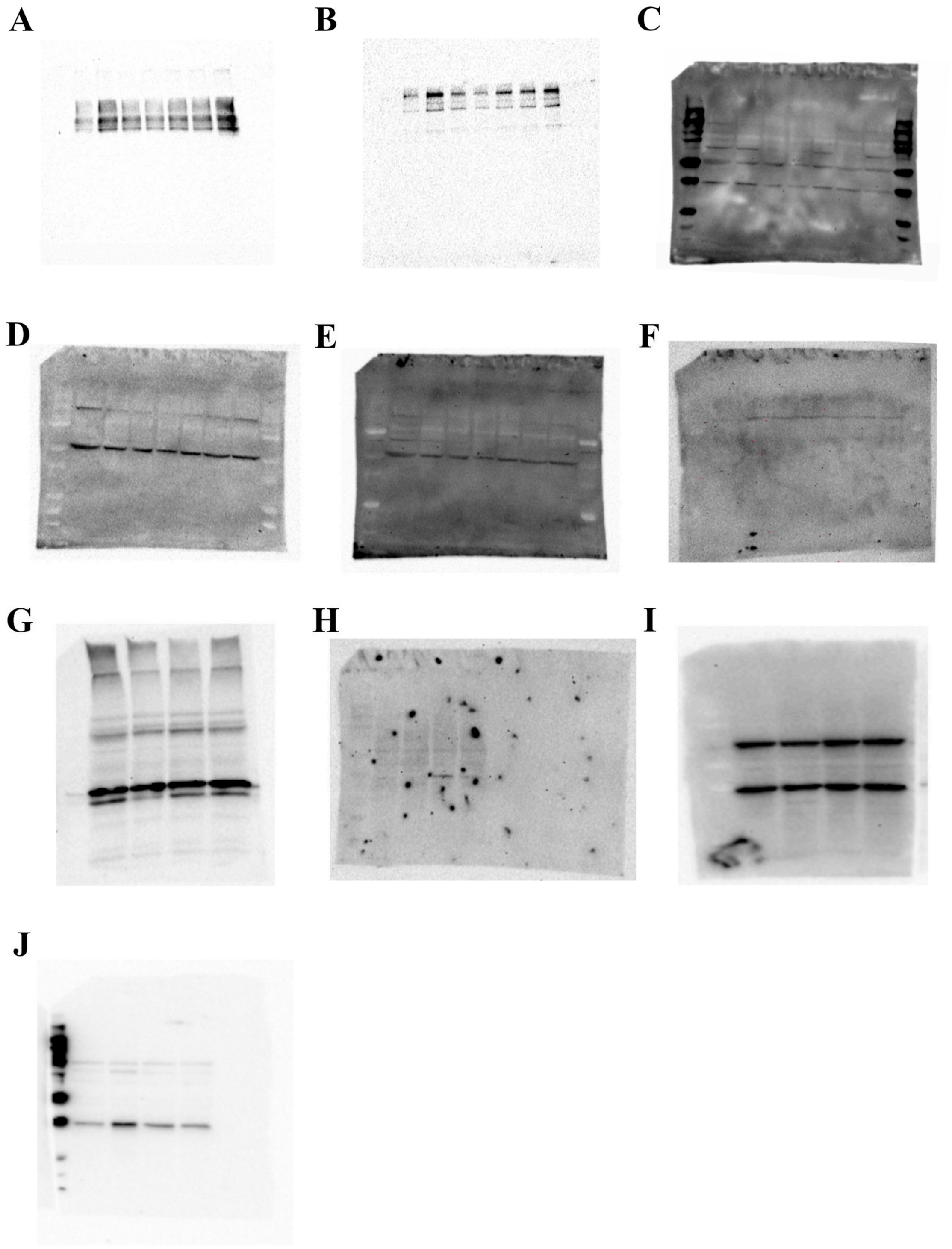

Figure S3: Full, uncropped Western blot membranes corresponding to the immunoblots presented in Figure 4B,D. Representative membranes are shown for (A) STAT1, (B) TBK1, (C) IKK, (D) IRF3, (E) NF-kB, (F) cGAS, (G) STING, (H) p-STING, (I) GAPDH, and (J) p-IRF3.

References

37. Maeß MB, Sendelbach S, Lorkowski S. Selection of reliable reference genes during THP-1 monocyte differentiation into macrophages. BMC Mol Biol (2010) 11:90. doi: 10.1186/1471-2199-11-90

59. Li M, Li X, Wang E, Luo E. Upregulation of Toll-like receptor 2 gene expression by acetylation of AP-2 alpha in THP-1 cells, a human monocytic cell line. Int J Biochem Cell Biol (2013) 45:1594–1599. doi: 10.1016/j.biocel.2013.05.003

60. Bisht A, Dickens M, Rutherfurd-Markwick K, Thota R, Mutukumira AN, Singh H. Chlorogenic Acid Potentiates the Anti-Inflammatory Activity of Curcumin in LPS-Stimulated THP-1 Cells. Nutrients (2020) 12:2706. doi: 10.3390/nu12092706

61. Trinh T-A, Hoang TX, Kim JY. All-trans retinoic acid increases NF-κB activity in PMA-stimulated THP-1 cells upon unmethylated CpG challenge by enhancing cell surface TLR9 expression. Mol Cell Biochem (2020) 473:167–177. doi: 10.1007/s11010-020-03817-4

62. Peng Y, Chen Y, Ma J, Zhou W, Wanxg Y, Wang Y, Zheng H, Shi W. Role and mechanism of the Dectin 1 mediated Syk/NF κB signaling pathway in Talaromyces marneffei infection. Exp Ther Med (2021) 23:84. doi: 10.3892/etm.2021.11007

63. Chen J, Jia G, Lv X, Li S. Type 1 Diabetes Mellitus-Related circRNAs Regulate CD4+ T Cell Functions. Biomed Res Int (2022) 2022: doi: 10.1155/2022/4625183

64. Mao L, Zhang L, Li H, Chen W, Wang H, Wu S, Guo C, Lu A, Yang G, An L, et al. Pathogenic Fungus Microsporum canis Activates the NLRP3 Inflammasome. Infect Immun (2014) 82:882–892. doi: 10.1128/IAI.01097-13

65. Cirmi S, Maugeri A, Russo C, Musumeci L, Navarra M, Lombardo GE. Oleacein Attenuates Lipopolysaccharide-Induced Inflammation in THP-1-Derived Macrophages by the Inhibition of TLR4/MyD88/NF-κB Pathway. Int J Mol Sci (2022) 23:1206. doi: 10.3390/ijms23031206

66. Chanput W, Mes J, Vreeburg RAM, Savelkoul HFJ, Wichers HJ. Transcription profiles of LPS-stimulated THP-1 monocytes and macrophages: a tool to study inflammation modulating effects of food-derived compounds. Food Funct (2010) 1:254. doi: 10.1039/c0fo00113a
